# Effects of expertise on football betting

**DOI:** 10.1186/1747-597X-7-18

**Published:** 2012-05-11

**Authors:** Yasser Khazaal, Anne Chatton, Joël Billieux, Lucio Bizzini, Grégoire Monney, Emmanuelle Fresard, Gabriel Thorens, Guido Bondolfi, Nady El-Guebaly, Daniele Zullino, Riaz Khan

**Affiliations:** 1 Geneva University Hospitals, Grand-pré 70 C, 1206 Geneva, Switzerland; 2 Psychological Sciences Research Institute, Catholic University of Louvain, Louvain-La-Neuve, Belgium; 3 Addiction Division, University of Calgary, Calgary, AB, Canada

**Keywords:** Betting, Sport betting, Sport, Football, Gambling, Addiction

## Abstract

**Background:**

Football (soccer) is one of the most popular sports in the world, including Europe. It is associated with important betting activities. A common belief, widely spread among those who participate in gambling activities, is that knowledge and expertise on football lead to better prediction skills for match outcomes. If unfounded, however, this belief should be considered as a form of “illusion of control.” The aim of this study was to examine whether football experts are better than nonexperts at predicting football match scores.

**Methods:**

Two hundred and fifty-eight persons took part in the study: 21.3% as football experts, 54.3% as laypersons (non-initiated to football), and 24.4% as football amateurs. They predicted the scores of the first 10 matches of the 2008 UEFA European Football Championship. Logistic regressions were carried out to assess the link between the accuracy of the forecasted scores and the expertise of the participants (expert, amateur, layperson), controlling for age and gender.

**Results:**

The variables assessed did not predict the accuracy of scoring prognosis (R^2^ ranged from 1% to 6%).

**Conclusions:**

Expertise, age, and gender did not appear to have an impact on the accuracy of the football match prognoses. Therefore, the belief that football expertise improves betting skills is no more than a cognitive distortion called the “illusion of control.” Gamblers may benefit from psychological interventions that target the illusion of control related to their believed links between betting skills and football expertise. Public health policies may need to consider the phenomenon in order to prevent problem gambling related to football betting.

## Introduction

Football is one of the most popular sports in the world, Europe included. It is associated with important monetary transactions and financial sponsoring [1].

Sports betting is associated with pathological gambling [2] and is widely available on the Internet [3], one of the most important means for seeking general, medical, and gambling information [4,5]. There, one can find messages such as “To win at sports betting, you have to prognosticate correctly. Don’t forget that a sports bet is not the lotto. Sport is not only a question of chance, far from it. To place your bet efficiently, you must learn about football as a sport and follow a minimum of its championships.”

Football competition is, unmistakably, a sport based on a high level of training and specific skills. This assertion may lead to the belief that football knowledge and expertise will allow better prediction of match scores. If unfounded, however, this belief should be considered a form of “illusion of control.” This term was defined by Langer [6] as “an expectancy of a personal success probability inappropriately higher than the objective probability would warrant.” This type of distorted thinking was considered a major factor in gambling persistence and severity [7,8], and led to the development of cognitive restructuring therapies for pathological gamblers [6].

As suggested by Cantinotti, Ladouceur, and Jacques [9], to a certain degree, the utility of sport expertise in sport betting cannot be fully ruled out. For example, it was previously found that factors such as the home field advantage, team rankings, most recent results of teams, and injuries of key players significantly affect game results [10-14]. It was then suggested that skills could be helpful when betting on sports events [15].

Probably in connection with these considerations in sport and football betting, defeats have been shown to be more often discussed than wins [16] and were commonly attributed to unlikely or random events [5] or were considered a “near win” [11], whereas wins were attributed to skills in selecting the victorious players. This interpretation probably contributes to an overestimation of betting skills [5].

It would be relevant to determine whether expertise is essential for determining game scores. If this were not the case, the alleged skills in sports betting could be regarded as no more than a manifestation of the illusion of control, as observed in most gambling activities.

With several exceptions, such as horse betting [17] and hockey [7], the relation between gamblers’ skills and betting outcomes has been rarely studied. Studies that evaluated gambling skills rather than the role of expertise in sports for betting activities showed that monetary gains from gambling skills were not significantly higher than would have occurred by chance. Because of the wide popularity of football and football betting, it seems important from a public health policy perspective to assess the links between football expertise and prediction of match results.

The present study examined whether football experts were better than non-experts for predicting the scores of the first 10 matches of the 2008 UEFA European Football Championship.

## Methods

### Procedure

During the 3 weeks prior to the beginning of the first match of the 2008 UEFA European Football Championship, a questionnaire was completed anonymously by 258 study participants recruited through local advertising and direct contact of football professionals (players, handlers, and referees) and sports reporters. The questionnaire assessed professional and amateur activity in relation to football. It also included five questions (Table 1) related to the degree of football interest (questions 1, 2, and 3), the degree of belief in the link between a good knowledge of football teams and accuracy of match-related prognoses (question 4), and sport betting habits (question 5). In addition, participants predicted outcomes for the first 10 matches of the 2008 UEFA European Football Championship.

**Table 1:** Questions related to football and sport betting

**Tick below the answer that mostly corresponds to you (only one answer possible)**					
	**Not at all**	**A bit**	**Fairly**	**Extremely**	
1) I am interested in football.					
2) I am going to follow the Euro 2008.					
3) I am a great fan of a team.					
4) I think that a good knowledge of the teams allows me to predict with accuracy the match score results.					
5) I often make sports bets (with monetary bets).	Never	Seldom	Quite often	Very often	Every time it’s possible

Participants were classified as being in one of three categories:

(a) “Experts”: The experts are professional or semiprofessional football players, coaches, or football sport journalists whose work was related to the 2008 UEFA European Football Championship.

(b) “Amateurs”: These participants have an amateur link with football (e.g., amateur referee) and/or play football as amateurs.

(c) “Laypersons”: This group has neither professional nor amateur connections.

The forecasts were analyzed for winning accuracy (accuracy of the prognosis: winning team 1, winning team 2, or draw) and score accuracy (good score prediction).

### Analyses

Statistical analyses were performed with SPSS for Windows (version 15.0). An initial exploratory analysis involved the calculation of proportions, as well as means and standard deviation of the outcome values. Spearman correlations with Bonferroni’s correction (*p* = 0.05/4 since four correlations were analyzed; *p* = 0.0125) were carried out to assess the links between each of the first four questions related to football interests and the fifth related to sports betting (Table 1). Moreover, one-way analyses of variance (ANOVAs) were performed to compare the distribution of the mean numbers of correct outcomes and correct score predictions as dependent variables with regard to the above-cited first four questions as factors, adjusting for multiple pairwise comparisons.

We also used a paired samples *t*-test to test whether gamblers had a greater number of correct outcomes than chance when forecasting the results of the games. Indeed, by chance, that is to say in the absence of any information, the probability of a gambler predicting 7 correct outcomes out of 10 games (0.016; the exact formula for the binomial distribution is given by $p\left(x\right)={(}_{x}^{n})p^{x}{\left(1-p\right)}^{n-x}$, where *x* is the number of successes and *n* the number of trials) is not the same as the probability of predicting 7 outcomes out of 10, given all the information in the bettor’s possession (0.7; given by the assumption: 7 correct outcomes out of 10 games). This last probability, referred to as conditional probability, means that before making a choice, the bettor will take into account all relevant information at their disposal. In addition to each participant’s observed probability of making the right bet, one may compare this to the expected probability based on chance.

Finally, a binary logistic regression for each of the 10 matches was done to predict the accuracy of the scores (correct vs. incorrect score) with the participants’ expertise categories (expert, amateur, or layperson) as predictor, controlling for age and gender (female vs. male). For the categorical factors “gender” and “expertise,” the reference groups were levels 2 and 3, respectively.

After checking for multicollinearity and outliers, we assessed the goodness of fit of these logistic models by considering the following:

The classification table of the intercept-only model (baseline or null model) with that of the full model, where a significant improvement should be expected over the null model.

The Nagelkerke R-square statistic with all the independent variables. This statistic attempts to quantify the proportion of explained variation in the logistic regression.

The statistical tests of the predictors, using the Wald chi-square statistics. P-values less than 0.05, or alternatively, confidence intervals that exclude the “1” value, are suggestive of significant predictions.

## Results

Two hundred and fifty-eight persons participated in the study (57% were men; mean age: 36.6 years ± 11.2). Fifty-five (21.3%) were classified as football experts, 140 (54.3%) as laypersons, and 63 (24.4%) as amateurs.

Answers to the five questions in the questionnaire are reported in Table 2. After Bonferroni’s correction was performed, the Spearman correlation showed significant associations between the first three questions related to football interest and sports betting (Spearman’s r = 0.49, r = 0.43, r = 0.41; *p* < 0.0005, respectively). Sports betting appeared to be associated with football interest. There was no correlation found between question 4 (believed role of football expertise for prognosis skills) and sports betting.

**Table 2:** **Distribution of participants’ answers to the five questions reported in Table**1

**N = 258 participants**	**%**
1) I am interested in football	
-	25.4
not at all	
-	26.3
a bit	
-	22.3
fairly	
-	26.0
extremely	
2) I am going to follow the Euro 2008	
-	19.5
not at all	
-	27.1
a bit	
-	23.6
fairly	
-	29.8
extremely	
3) I am a great fan of a team	
-	46.5
not at all	
-	15.9
a bit	
-	17.8
fairly	
-	19.8
extremely	
4) I think that a good knowledge of the teams allows me to predict with accuracy the match score results	
-	27.2
not at all	
-	36.8
a bit	
-	30.2
fairly	
-	5.8
extremely	
5) I often make sports bets (with monetary bets)	
-	81.0
never	
-	14.3
seldom	
-	3.5
quite often	
-	1.2
very often	
every time it’s possible	0.0

**Figure 1:**
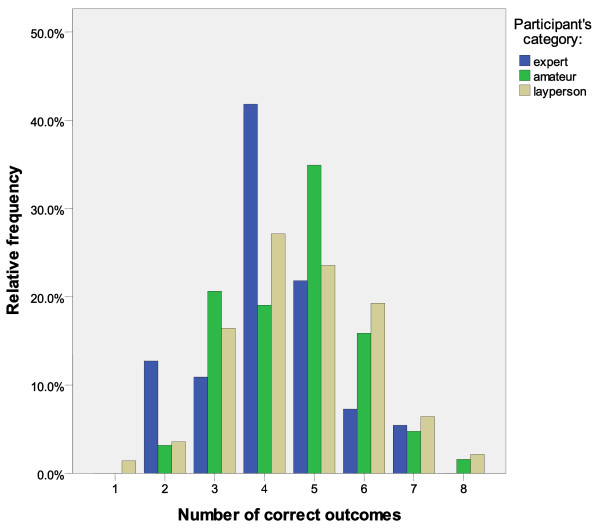
Distribution of relative frequencies of correct outcomes.

The numbers and percentages of accurate outcomes and scores by category of participants are reported in Table 3. The mean number of correct outcome or score prognoses and the relative frequency of the distribution of correct outcomes by category of participants are reported in Table 4 and Figure 1, respectively.

**Table 3:** Number and percentage of correct outcomes and scores by categories of participants and by match

	**Correct outcomes, n (%)**			**Correct scores, n (%)**		
**Match**	**Expert (n = 55)**	**Amateur (n = 63)**	**Layperson (n = 140)**	**Expert (n = 55)**	**Amateur (n = 63)**	**Layperson (n = 140)**
1. Switzerland – Czech Republic	11 (20)	23 (36.5)	38 (27.1)	1 (1.8)	4 (6.3)	10 (7.1)
2. Portugal – Turkey	45 (81.8)	47 (74.6)	105 (75)	14 (25.5)	15 (23.8)	18 (12.9)
3. Austria – Croatia	41 (74.5)	35 (55.6)	69 (49.3)	8 (14.5)	8 (12.7)	18 (12.9)
4. Germany – Poland	35 (63.6)	50 (79.4)	119 (85.0)	9 (16.4)	17 (27.0)	23 (16.4)
5. Romania – France	8 (14.5)	8 (12.7)	24 (17.1)	1 (1.8)	1 (1.6)	5 (3.6)
6. Netherlands – Italy	6 (10.9)	11 (17.5)	16 (11.4)	0 (0)	0 (0)	2 (1.4)
7. Spain – Russia	33 (60)	45 (71.4)	95 (67.9)	1 (1.8)	0 (0)	3 (2.1)
8. Greece – Sweden	13 (23.6)	18 (28.6)	44 (31.4)	2 (3.6)	4 (6.3)	8 (5.7)
9. Czech Republic – Portugal	27 (49.1)	36 (57.1)	98 (70)	6 (10.9)	4 (6.3)	13 (9.3)
10. Switzerland – Turkey	10 (18.2)	17 (27)	37 (26.4)	2 (3.6)	6 (9.5)	8 (5.7)

**Table 4:** Comparison of mean number (standard deviation) of correct outcomes and scores exactly predicted by each group of participants

	**Expert**	**Amateur**	**Layperson**
Mean number of correct outcomes	4.16 (1.27)	4.60 (1.29)	4.62 (1.41)
Mean number of correct score predictions	0.80 (0.80)	0.94 (0.86)	0.77 (0.83)

The ANOVAs that were used to compare the distribution of the mean numbers of correct outcomes and correct score predictions with regard to the first four questions showed a statistical significant between-group difference for the mean number of correct outcomes (F_(3,253)_ = 2.8 and *p* = 0.04) in question 4 (“I think that a good knowledge of the teams allows me to predict with accuracy the match score results”). But after adjusting for multiple comparisons, this difference was no longer significant. No significant difference before or after adjustment was observed for the other three questions.

The paired sample *t*-test that was used to evaluate whether gamblers had a greater number of correct outcomes than chance when forecasting the results of the games showed a statistical significant difference (t = 39.15 and *p* < 0.0005). We conclude from the data that the bettors were more accurate in their predictions than chance.

The logistic regressions, which were done to test the research hypothesis, yielded poor results. The classification table of the full models showed no improvement over the baseline models, meaning that the classification rates were exactly the same in both situations (Table 5, column 2). The Nagelkerke R-square measures ranged from 1% to 6%, leading us to conclude that these models were not useful in explaining the outcome variable (Table 5, column 3). Finally, the predictor variable “expertise” (Table 5, column 4) did not prove significant except for the model involving the game Germany-Poland (*p* = 0.03, odds ratio = 2.57 and 95% CI for odds ratio = [1.08; 6.14]). This result means that compared with a layperson, being an amateur increases the likelihood of accurate score prediction by 2.57, assuming that the other factors in the model are held constant. There was no significant difference between experts and laypersons since the confidence interval includes “1” (exact results not shown).

**Table 5:** Evaluation of model’s goodness of fit

**Match**	**Status of correct cases classified in full model compared with null modelM**	**Nagelkerke’s R-squared (in %)**	**Status of predictor variable “expertise” in the regression**
1. Switzerland – Czech Republic	Unchanged	4	Not significant
2. Portugal – Turkey	Unchanged	6	Not significant
3. Austria – Croatia	Unchanged	1	Not significant
4. Germany – Poland	Unchanged	3	Significant
5. Romania – France	Unchanged	5	Not significant
6. Netherlands – Italy	Unchanged	*	*
7. Spain – Russia	Unchanged	*	*
8. Greece – Sweden	Unchanged	5	Not significant
9. Czech Republic – Portugal	Unchanged	5	Not significant
10. Switzerland – Turkey	Unchanged	4	Not significant

*Maximum iterations reached and final solution not found because group sample size too small.

It is worthwhile noting that no expert was able to correctly predict more than seven outcomes and no participant more than eight (Figure 1).

## Discussion

In the present study, the results of the logistic regressions, although poor, were consistent across matches. Experts do not appear to be better than non-experts at predicting football match scores. Similarly, ANOVA results indicated that the average number of correct outcomes with respect to accurate scores were not significantly different across the four conditions (first four questions in Table 1). The belief that expertise is useful for sports gamblers seems to be simply an illusion of control.

By chance alone, the probability of someone predicting 10 correct outcomes (first winning team, second winning team, or draw) out of 10 games is estimated to be $\frac{1.7}{100,000}$, i.e., $P\left(X=10\right)=\frac{10!}{10!0!}\times {\frac{1}{3}}^{10}\times {\frac{2}{3}}^{0}$. The exact formula for the binomial distribution is given by $p\left(x\right)={(}_{x}^{n})p^{x}{\left(1-p\right)}^{n-x}$, where χ is the number of successes and *n* the number of trials. This is an interesting probability for the sports betting business, which mostly offers big monetary winnings on a combination of match results. Thus, in consideration of this probability and the lack of impact of expertise on football betting outcomes, sports betting appears to be nothing other than a game of chance, as suggested by other studies [7,15].

As reported elsewhere [18], sport interest, rather than the athlete’s status [19], is possibly linked to sport betting. The finding of a lack of association between sports bets and the belief assessed in question 4 (“I think that a good knowledge of the teams allows me to predict with accuracy the match score results”) may result from the present sample being participants from the community and not problem or pathological gamblers. About 6% of the subjects did, however, consider this declaration as extremely correct and more than 30% as fairly correct, showing the wide diffusion of such beliefs.

One possible limitation of the present study is that it was not carried out as a real gambling condition. The results should be then taken with caution. Further studies may include measures of gambling-related cognitions (e.g., fallacy, superstitious beliefs, biased evaluation of outcomes), participants’ betting behaviors and habits, and more detailed measures of expertise and self-confidence related to a sense of expertise, as suggested by wagering models [20]. Further studies may also include betting related to other sport activities.

Another limitation was the small sample of games surveyed and the non-random selection of these games, which resulted in a non-probability sample. Out of all matches that were played during the 2008 UEFA European Football Championship, only the first 10 were selected for analysis. The possibility that, by pure chance, the games selected happened to be more or less predictable than the standard ones should not be ignored.

Finally, the logistic regression models set up to predict the accuracy of the number of goals scored by each team may have failed to take account of other possible significant predictors, such as the quality of the teams (ability to attack and/or to defend), the team’s league position at the time of playing, and the “home effect” (advantage in playing at home). The absence of these potential predictors may explain the small predictive power of our models.

## Conclusion

Expertise, gender, and age did not have an impact on the accuracy of the football match prognoses. Consequently, the belief that football expertise improves betting skills seems to be a cognitive distortion.

Clinicians may inform gamblers about the limited help of football expertise in match-outcome predictions and the relative fallacy of commercial advertisements for sport betting, such as “….a sports bet is not the lotto… to place your bet efficiently, you must learn about football as a sport and follow a minimum of its championships.” Gamblers may benefit from psychological interventions that target the illusion of control related to their believed links between betting skills and football expertise. Furthermore, public health prevention policies may need to consider the present results in order to prevent problem gambling related to football betting.

## Competing interests

The authors declare that they have no competing interests.

## Authors’ contributions

YK and DZ participated in the design of the study. YK and NG drafted the manuscript. AC performed the statistical analysis. JB, LB, GM, EF, GT, RK, and GB participated in recruitment and data collection. All authors read and approved the final manuscript.

## References

[B1] Maher A Wilson N Signal L Thomson G Patterns of sports sponsorship by gambling, alcohol and food companies: an Internet survey BMC Publ Health 2006 6 95 10.1186/1471-2458-6-95 PMC1459130 16608525

[B2] Peltzer K Mabilu MG Mathoho SF Nekhwevha AP Sikhwivhilu T Sinthumule TS Trauma history and severity of gambling involvement among horse-race gamblers in a South African gambling setting Psychol Rep 2006 99 472 476 17153816 10.2466/pr0.99.2.472-476

[B3] Labrie RA Laplante DA Nelson SE Schumann A Shaffer HJ Assessing the playing field: a prospective longitudinal study of Internet sports gambling behavior J Gambl Stud 2007 23 347 362 10.1007/s10899-007-9067-3 17574522

[B4] Khazaal Y Chatton A Cochand S Jermann F Osiek C Bondolfi G Zullino D Quality of web-based information on pathological gambling J Gambl Stud 2008 24 357 366 10.1007/s10899-008-9095-7 18373182

[B5] Coquard O Fernandez S Zullino D Khazaal Y A follow-up study on the quality of alcohol dependence-related information on the web Subst Abuse Treat Prev Policy 2011 6 13 10.1186/1747-597X-6-13 21663650 PMC3121592

[B6] Langer E The illusion of control J Pers Soc Psychol 1975 32 311 328

[B7] Toneatto T Blitz-Miller T Calderwood K Dragonetti R Tsanos A Cognitive distortions in heavy gambling J Gambl Stud 1997 13 253 266 10.1023/A:1024983300428 12913389

[B8] Tavares H Martins SS Lobo DS Silveira CM Gentil V Hodgins DC Factors at play in faster progression for female pathological gamblers: an exploratory analysis J Clin Psychiatry 2003 64 433 438 10.4088/JCP.v64n0413 12716246

[B9] Cantinotti M Ladouceur R Jacques C Sports betting: can gamblers beat randomness? Psychol Addict Behav 2004 18 143 147 15238056 10.1037/0893-164X.18.2.143

[B10] Boulier BL Stekler HL Are sports seedings good predictors?: an evaluation International J Forecasting 1999 15 83 91 10.1016/S0169-2070(98)00067-3

[B11] Vergin RC Sosik JJ No place like home: an examination of the home field advantage in gambling strategies in NFL football J Econ Bus 1999 51 21 31 10.1016/S0148-6195(98)00025-3

[B12] Carron AV Loughhead TM Bray SR The home advantage in sport competitions: Courneya and Carron's (1992) conceptual framework a decade later J Sports Sci 2005 23 4 395 407 10.1080/02640410400021542 16089184

[B13] Forrest D Simmons R Outcome uncertainty and attendance demand in sport: the case of English soccer J Royal Stat Soc: Series D, Statistician 2002 51 2 229 241 10.1111/1467-9884.00314

[B14] Forrest D Beaumont J Goddard J Simmons R Home advantage and the debate about competitive balance in professional sports leagues J Sports Sci 2005 23 4 439 445 10.1080/02640410400021641 16089188

[B15] Rogers P The cognitive psychology of lottery gambling: a theoretical review J Gambl Stud 1998 14 111 134 10.1023/A:1023042708217 12766438

[B16] Gilovich T Biased evaluation and persistence in gambling J Pers Soc Psychol 1983 44 1110 1126 6875803 10.1037//0022-3514.44.6.1110

[B17] Ladouceur R Giroux I Jacques C Winning on the horses: how much strategy and knowledge are needed? J Psychol 1998 132 133 142 10.1080/00223989809599154

[B18] Nelson TF LaBrie RA LaPlante DA Stanton M Shaffer HJ Wechsler H Sports betting and other gambling in athletes, fans, and other college students Res Q Exerc Sport 2007 78 271 283 10.5641/193250307X13082490461264 17941532

[B19] Weinstock J Whelan JP Meyers AW Watson JM Gambling behavior of student-athletes and a student cohort: what are the odds? J Gambl Stud 2007 23 13 24 10.1007/s10899-006-9043-3 17191145

[B20] Sierra J Hyman M In search of value: a model of wagering intentions J Marketing Theory Pract 2009 17 235 249 10.2753/MTP1069-6679170303

